# Pathogenic effects of *Halomonas* on cyanobacteria and biocontrol potential of a deep-sea *Bacillus* strain

**DOI:** 10.1128/aem.00199-26

**Published:** 2026-03-20

**Authors:** Xinyi Li, Meilin Yuan, Chaomin Sun, Shimei Wu

**Affiliations:** 1 College of Life Sciences, Qingdao University, Qingdao, China; 2 CAS Key Laboratory of Experimental Marine Biology, Institute of Oceanology, Chinese Academy of Sciences 53014, Qingdao, China; 3 Laboratory for Marine Biology and Biotechnology, Qingdao National Laboratory for Marine Science and Technology 474988 , Qingdao, China; Colorado School of Mines, Golden, Colorado, USA

**Keywords:** Cyanobacteria, *Limnospira fusiformis*, *Halomonas*, pathogen, biocontrol

## Abstract

*Limnospira fusiformis*, a nutritionally valuable cyanobacterium with significant biomanufacturing potential, faces critical challenges from pathogenic invasions. This study identified *Halomonas variabilis* 2-9 as the primary pathogen of *L. fusiformis* cultivation collapse, demonstrating broad-spectrum algicidal activity against multiple cyanobacterial species. Additionally, the algicidal substance produced by *H. variabilis* 2-9 was purified and identified as toxic dibutyl phthalate. At a concentration of 3 µg/mL, the purified algicidal substance caused 90.6% reduction in Fv/Fm within 24 h and 86% degradation of chlorophyll-*a* within 120 h. To be specific, Fv/Fm as the maximum photochemical quantum yield of photosystem II is a core indicator reflecting the photosynthetic activity and health status of spirulina. A sudden drop in Fv/Fm of spirulina indicates impaired function of Photosystem II. Therefore, the purified algicidal substance severely damaged the photosynthetic system of *L. fusiformis*. To mitigate the detrimental effects of *H. variabilis* 2-9, *Bacillus velezensis* L4 was isolated from deep-sea sediments. This strain produced an antimicrobial compound (C_31_H_42_O_2_) that specifically inhibited *H. variabilis* 2-9 without damaging *L. fusiformis*. In co-culture experiments, *B. velezensis* L4 reversed *Halomonas*-induced algal decay, demonstrating its potential as a biocontrol agent. These findings provide both mechanistic insights into *Halomonas*-mediated cyanobacterial pathogenesis and a practical solution for sustainable aquaculture management.

**IMPORTANCE:**

This study identifies *H. variabilis* 2-9 as a novel cyanobacterial pathogen that produces hazardous compound dibutyl phthalate (DBP), causing severe damage to *L. fusiformis* and exhibiting broad-spectrum algicidal activity against other cyanobacteria. The discovery of DBP-mediated pathogenesis provides crucial insights into microbial threats to aquaculture systems. Significantly, we demonstrate that *B. velezensis* L4, isolated from deep-sea environments, serves as an effective biocontrol agent through the production of a selective antimicrobial compound that specifically targets *H. variabilis* 2-9 without harming *L. fusiformis*. These findings offer both fundamental understanding of cyanobacterial disease mechanisms and a practical, sustainable solution for algal disease management.

## INTRODUCTION

Cyanobacteria, commonly known as blue-green algae, have played a pivotal role in shaping early biogeochemical cycles and planetary development. As the most primitive photosynthetic organisms, they are the only prokaryotic organisms capable of performing oxygenic photosynthesis (1). Meanwhile, cyanobacteria also play an important role in the biogeochemical cycles of carbon and nitrogen (2–4). These organisms are widely distributed across terrestrial, freshwater, wastewater, and marine environments due to their remarkable morphological variability and physiological adaptability (5). However, their rapid growth can easily lead to harmful algal blooms when nutrient concentrations and water temperature conditions are favorable (6, 7). Algal blooms pose significant threats to ecosystems, socio-economic systems, and human health (8). Dense algal growth can cover the water surface, inhibiting gas and energy exchange between water and the atmosphere (9). The blooms also increase water turbidity, release mucilaginous substances, and produce foul odors, severely impairing water resource quality and utilization (10). Furthermore, certain cyanobacteria, such as *Microcystis*, produce and release harmful cyanotoxins, which can contaminate water sources and pose risks to aquatic and terrestrial organisms, including humans (11).

Cyanobacteria, beyond their ecological impact, have gained significant attention in biotechnology and industrial applications due to their ability to produce bioactive compounds including toxins, as well as substances with antibacterial, antiviral, anticancer, and immunomodulatory activities (12). *Limnospira* (previously classified as *Arthrospira*) is a recently established cyanobacterial genus comprising species such as *L. fusiformis*, *L. indica*, and *L. maxima* (13). Known for its environmental sustainability (14), *Limnospira* is widely used as a dietary supplement due to its diverse health benefits (15–17). It also has applications in nutraceuticals, pharmaceuticals, bioremediation, and industrial biomanufacturing (18). Marketed globally as “spirulina,” *Limnospira* is extensively cultivated for its high nutritional value, especially its protein content, and its rapid growth (19).

However, cyanobacterial cultivation is often associated with environmental contamination risks, particularly in open systems like ponds and lakes, which are vulnerable to pathogen invasions, including bacteria, fungi, and viruses (20–23). Pathogens employ diverse strategies to inhibit or lyse cyanobacteria, such as producing algicidal compounds (e.g., peptides, enzymes, or secondary metabolites), competing for nutrients, and engaging in direct physical interactions (24–26). A significant portion of research has focused on the algicidal bacteria to control harmful algal blooms. For example, *Morganella morganii* and *Microcystis aeruginosa* can secrete proteases or lipopeptides that degrade algal cells, showing potential for treating harmful blooms (27, 28). Despite extensive research on pathogens affecting bloom-forming cyanobacteria, much less is known about those impacting commercially cultivated species like *Limnospira*.

During cultivation, *L. fusiformis* is often contaminated by bacteria that thrive in saline-alkaline conditions, such as *Rhodobaca* sp. and *Salinispirillum* sp. (29). Specifically, *Halomonas* sp. can not only survive but also reproduce prolifically in alkaline environments, and it has been reported to exhibit algicidal effects (30). In addressing these pollution issues that arise in the cultivation of spirulina, various measures have been actively adopted. These include physical approaches, such as closed culture systems and post-separation purification, as well as chemical methods involving the use of antibiotics and chemical agents to modify the culture environment (31–33). However, physical control has the problem of high cost, and the excessive use of chemical agents often leads to side effects on the environment (34). In contrast, biological control presents a promising and environmentally friendly alternative for managing contamination.

In this study, a *Halomonas* strain was isolated from the diseased *L. fusiformis* collected from an industrial farm. To understand its pathogenicity, the algicidal substance and underlying mechanism were investigated. Additionally, a *Bacillus* strain with strong inhibitory activity against the pathogenic *Halomonas* strain was isolated from the deep-sea sediments, showing promising potential as a biocontrol agent for *L. fusiformis* disease, and the active substance produced by the *Bacillus* strain was also purified and identified.

## RESULTS

### Isolation and identification of *L. fusiformis* pathogen

**Fig 1:**
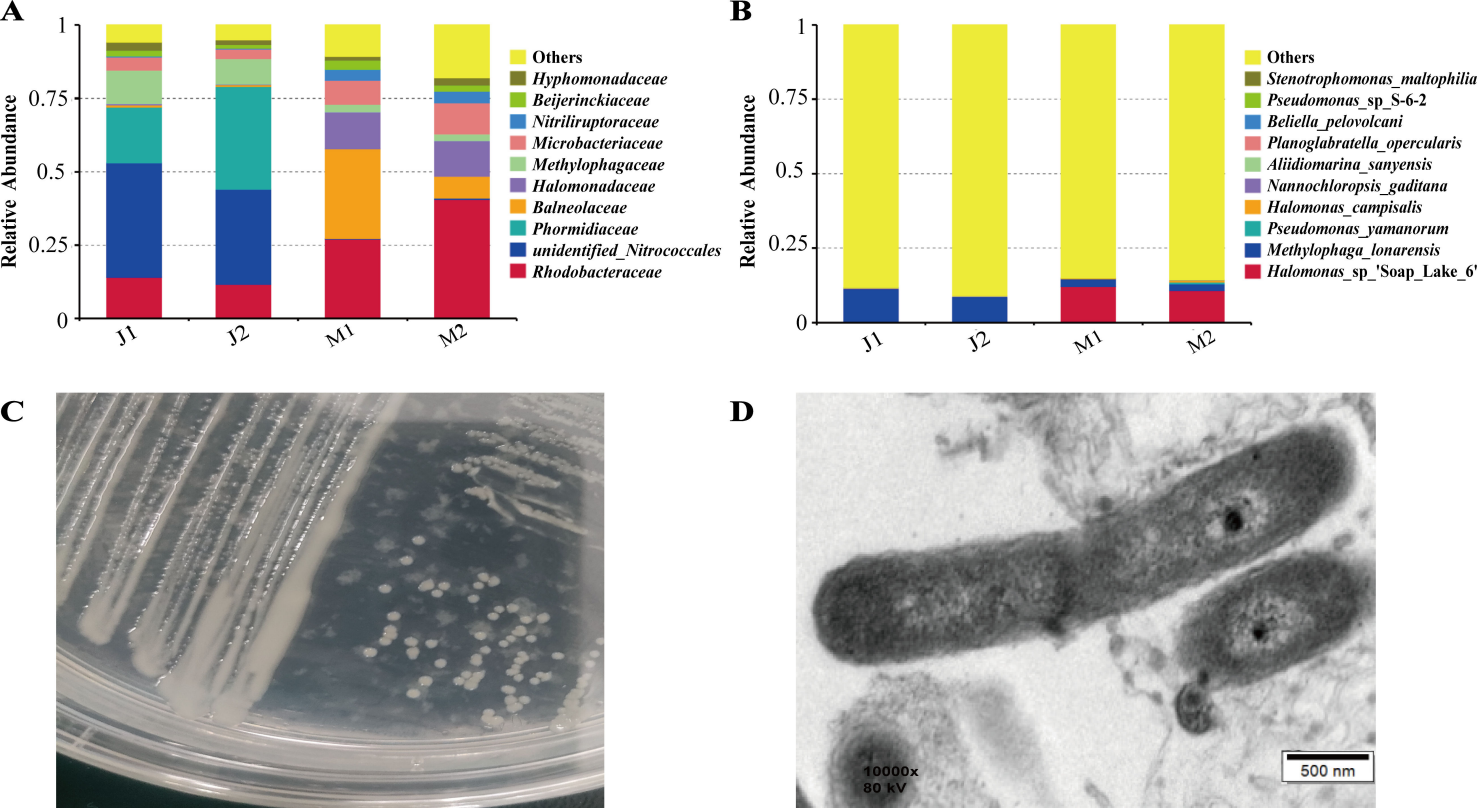
Isolation and identification of *L. fusiformis* pathogen. (**A**) Relative abundance of the top 10 microbial families and (**B**) top 10 microbial species in healthy J samples and diseased M samples. (**C**) Growth of *H. variabilis* 2-9 colonies on 2216E medium. (**D**) Morphology of *H. variabilis* 2-9 observed under TEM.

To identify potential pathogen of *L. fusiformis*, biodiversity analysis was performed using 16S rRNA gene sequencing between healthy and diseased samples. The results revealed that the Halomonadaceae family was a dominant microbial group, comprising 12.6% and 12.1% of the total microbial communities in the diseased samples M1 and M2, respectively. In contrast, its presence in healthy samples J1 and J2 was minimal, accounting for only 0.47% and 0.15% (Fig. 1A), respectively. Additionally, as shown in Fig. 1B, *Halomonas* sp. represented 12% and 10.7% of the microbial populations in diseased samples M1 and M2, respectively, but only 0.15% and 0.04%, respectively, in healthy samples J1 and J2. These findings indicate a notable increase in the relative abundance of *Halomonas* in diseased *L. fusiformis*, strongly suggesting it as the primary pathogenic bacterium.

Following the identification of *Halomonas* as the potential pathogenic bacterium, bacterial strains were isolated and purified from the diseased *L. fusiformis* culture. Among the 11 *Halomonas* strains obtained, strain 2-9 was selected for further investigation due to its pronounced algicidal activity against *L. fusiformis*. Further characterization of strain 2-9 revealed that it forms regular, smooth, and milky-white colonies on 2216E solid medium (Fig. 1C). Transmission electron microscopy (TEM) analysis revealed that the strain exhibits a regular oval morphology, with an average length of about 2–3 μm (Fig. 1D). The 16S rRNA gene sequence analysis revealed that strain 2-9 shared 99.66% sequence similarity with *Halomonas variabilis* strain HTG7 in the NCBI database. In the phylogenetic tree, strain 2-9 formed a robust clade with this reference strain (Fig. S1). Therefore, it was conclusively identified as *H. variabilis* and was designated as *H. variabilis* 2-9. For the 10 isolated *Halomonas* strains, according to 16S rRNA sequence analysis, 6 strains exhibit high similarity to *Halomonas qaidamensis*, while 2 strains are closely related to *Halomonas alkaliantarctica*. Additionally, one strain shows significant similarity to *Halomonas nitrilica*, and one strain is closely aligned with *Halomonas hydrothermalis* (Table S1).

### Algicidal activity of *H. variabilis* 2-9

**Fig 2:**
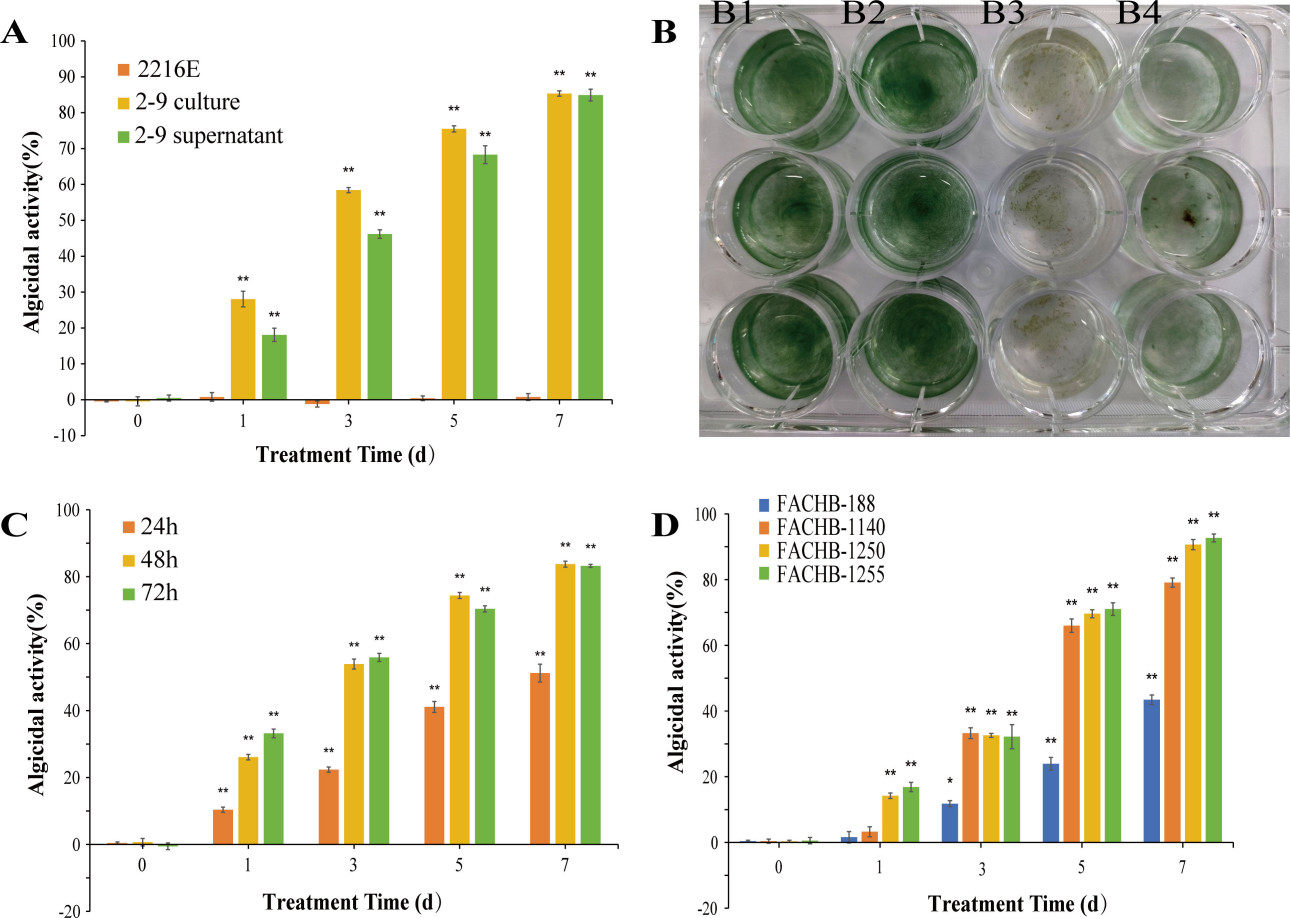
Algicidal activity of *H. variabilis* 2-9 against algae at a concentration of 2% (vol/vol). (**A**) Algicidal activity of *H. variabilis* 2-9 culture and supernatant on *L. fusiformis*. (**B**) Photograph of *L. fusiformis* on the 7th day: Healthy sample (B1), control with 2216E (B2), treatment with *H. variabilis* 2-9 culture (B3), and treatment with *H. variabilis* 2-9 supernatant (B4). (**C**) Algicidal activity of *H. variabilis* 2-9 supernatant at different fermentation time. (**D**) Algicidal activity of *H. variabilis* 2-9 supernatant against *Anabaena* sp. FACHB-188 and FACHB-1140, *Dolichospermum* sp. FACHB-1250, *Dolichospermum flos-aquae* FACHB-1255. All error bars indicate the standard error of three replicates. *, *P* < 0.05 **, *P* < 0.01 compared with the control group.

To explore the algicidal mode, the algicidal effects of both the *H. variabilis* 2-9 cell culture and its cell-free supernatant were evaluated independently. As shown in Fig. 2A, the algicidal activity of the *H. variabilis* 2-9 culture or its cell-free supernatant increased progressively with treatment duration. Specifically, after 7 days of treatment, the algicidal activity reached 85.3% for the culture and 84.9% for the cell-free supernatant. In contrast, no algicidal activity was detected in the control group treated with 2216E medium. Microscopic observations indicated that algal bodies treated with strain 2-9 exhibited a gray discoloration and showed signs of fracture and dissolution. In contrast, the control group that did not receive 2-9 treatment remained green and displayed healthy growth (Fig. S2). These results indicate that both the supernatant and the culture exhibit significant algicidal activity against *L. fusiformis*, suggesting that the algicidal mode of *H. variabilis* 2-9 is likely mediated by extracellular substances present in the culture supernatant. As depicted in Fig. 2B, a notable decrease in *L. fusiformis* density was observed in wells treated with either the culture or the cell-free supernatant of *H. variabilis* 2-9, accompanied by visible algal decay. Based on the statistical analysis and visual observations, it can be concluded that *H. variabilis* 2-9 produces and releases algicidal compounds that significantly inhibit the growth of *L. fusiformis*.

To determine the optimal phase of algicidal substance production by *H. variabilis* 2-9, the strain was fermented for 24, 48, and 72 h respectively, and the algicidal activity of the corresponding cell-free supernatant was detected. As shown in Fig. 2C, the algicidal activity of the supernatants increased with fermentation time. Specifically, the supernatants from 48 and 72 h of fermentation exhibited similar algicidal efficacy, achieving 83.7% and 83.2% inhibition of *L. fusiformis* after 7 days of treatment, respectively. In contrast, the supernatant from 24 h of fermentation showed lower activity, reaching only 51.1% inhibition. These results indicate that *H. variabilis* 2-9 primarily synthesizes algicidal compounds around the 48-h mark of fermentation.

To assess the pathogenicity spectrum of *H. variabilis* 2-9, its algicidal activity was tested against four different algal species. As illustrated in Fig. 2D, the supernatants displayed varying levels of algicidal efficacy across the tested species. Specifically, *Anabaena* sp. FACHB-188 showed 43% inhibition after 7 days, while *Anabaena* sp. FACHB-1140 exhibited 79% inhibition. *Dolichospermum* sp. FACHB-1250 achieved 90% inhibition, and *Dolichospermum fos-aguae* FACHB-1255 reached 92% inhibition. These findings indicate that *H. variabilis* 2-9 not only targets *L. fusiformis* but also demonstrates significant algicidal activity against a wide range of other cyanobacterial species.

### Isolation and identification of algicidal substances

**Fig 3:**
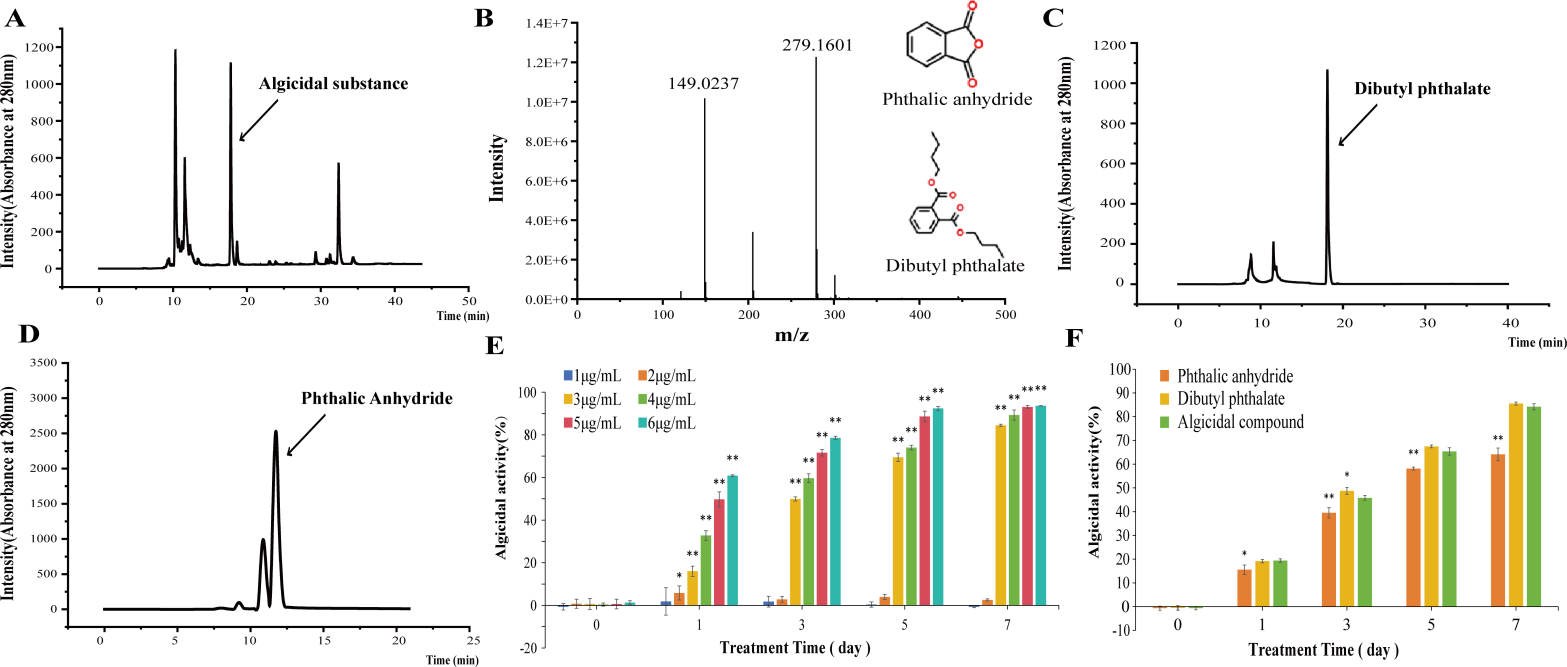
Purification and identification of the algicidal substance. (**A**) RP-HPLC analysis of the algicidal substance produced by *H. variabilis* 2-9. (**B**) LC-MS analysis of the fraction eluted at 17.803 min. (**C**) RP-HPLC analysis of commercial dibutyl phthalate. (**D**) RP-HPLC analysis of commercial phthalic anhydride. (**E**) Algicidal activity of different concentrations of the algicidal substance. (**F**) Algicidal activity of algicidal substance and corresponding commercial compounds at a concentration of 3 µg/mL. All error bars indicate the standard error of three replicates. *, *P* < 0.05 **, *P* < 0.01 compared with the control group.

To identify the algicidal compounds produced by *H. variabilis* 2-9, the supernatant was extracted with ethyl acetate, followed by methanol extraction, and the resulting crude extract was further purified using RP-HPLC. As shown in Fig. 3A and A fraction with a retention time of 17.803 min demonstrated significant algicidal activity. To determine the precise molecular mass of the active compound, this fraction was subjected to LC-MS analysis. As depicted in Fig. 3B, two distinct peaks were observed at *m*/*z* values of 149.0237 and 279.1601, corresponding to the single protonated forms ([M+H]^+^) of the compounds. The LC-MS analysis also suggested corresponding molecular formulas of C_8_H_4_O_3_ and C_16_H_22_O_4_. Given the structural similarity between these compounds and the fact that C_8_H_4_O_3_ corresponds to phthalic anhydride (PA), it is hypothesized that C_16_H_22_O_4_ represents a common derivative of PA, specifically dibutyl phthalate (DBP).

Due to the structural similarity between PA and DBP, their separation is challenging. To further identify the algicidal compounds produced by *H. variabilis* 2-9, we compared its liquid chromatography profiles and algicidal activities with those of commercial PA and DBP. As shown in Fig. 3C, under identical chromatographic conditions, the retention time of commercial DBP was about 18.1279 min, which is similar to that of the algicidal substance produced by *H. variabilis* 2-9, while the retention time of commercial PA is 11.6483 min (Fig. 3D).

To evaluate the algicidal efficacy of the substance produced by *H. variabilis* 2-9, we examined its dose-dependent effects on *L. fusiformis*. As shown in Fig. 3E, no significant algicidal effect was observed at a final concentration of 1 µg/mL. However, obvious algicidal effects were detected at concentrations of 3 µg/mL. The compounds exhibited dose-dependent activity, and inhibition rates reached 84.4%, 89.3%, 93.1%, and 93.6% at final concentrations of 3, 4, 5, and 6 µg/mL, respectively, after 7 days of treatment. Given the marked increase in efficacy at 3 µg/mL, this concentration was selected for subsequent experiments.

Subsequently, we assessed the algicidal activities of substances produced by *H. variabilis* 2-9, along with commercial PA and DBP, at a concentration of 3 µg/mL. As shown in Fig. 3F, both DBP and the purified algicidal substance exhibited comparable algicidal activity, inhibiting approximately 85% and 84% of algal growth after 7 days of treatment, respectively. In contrast, PA achieved only 64% inhibition at the same concentration and time point, which was significantly lower than that of the purified algicidal substance. Based on these results, we conclude that the primary algicidal component produced by *H. variabilis* 2-9 is DBP.

### Algicidal substance induced damage to the photosynthetic system of *L. fusiformis*

**Fig 4:**
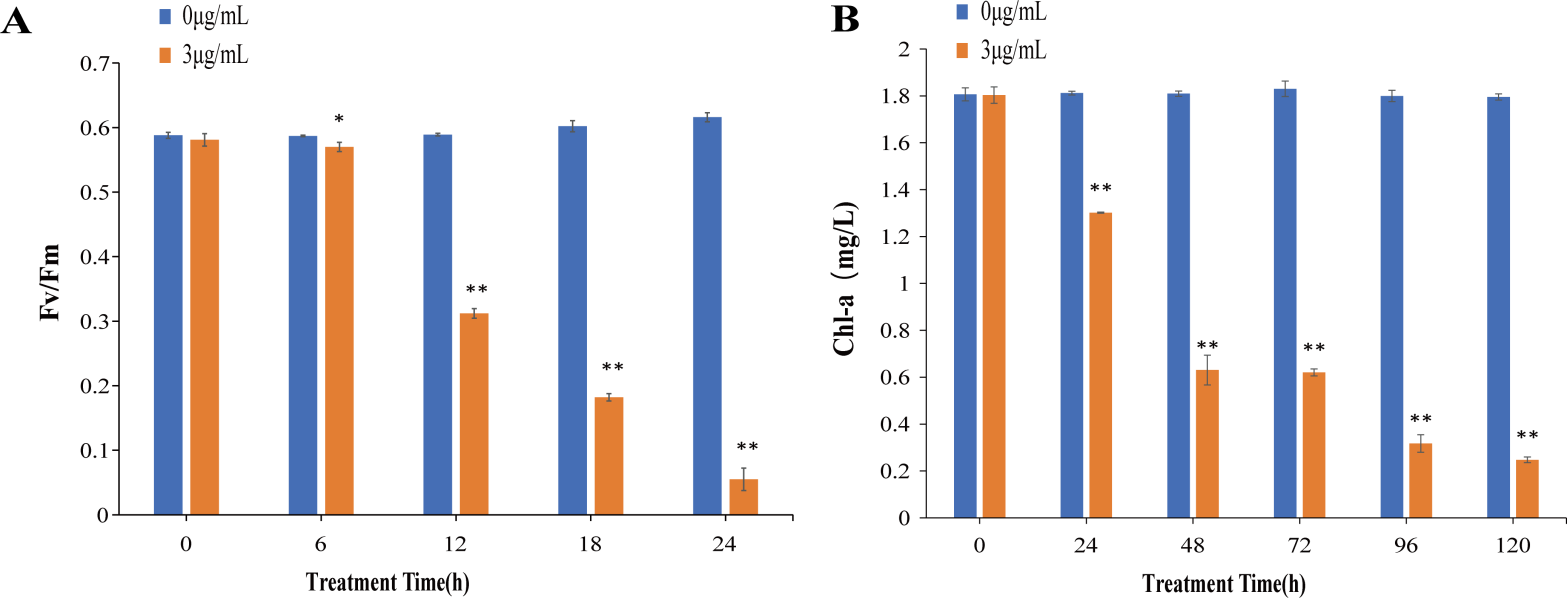
Effects of the algicidal substance on the photosynthetic system of *L. fusiformis* after treatment with 3 µg/mL of algicidal substance. (**A**) Fv/Fm ratio after treatment for 6, 12, 18, and 24 h. (**B**) Chl-a content after treatment for 0, 24, 48, 72, 96, and 120 h. All error bars indicate the standard error of three replicates. *, *P* < 0.05 **, *P* < 0.01 compared with the control group.

To evaluate the impact of the algicidal substances on the photosynthetic apparatus of *L. fusiformis*, two key parameters were measured: Fv/Fm and the concentration of chlorophyll a (Chl-a). Photosynthetic efficiency in algal cells relies on the capture of light energy, which is subsequently converted into chemical energy through photosynthesis. The Fv/Fm ratio serves as a critical indicator of the maximum photochemical efficiency of PSII, reflecting the health and functionality of the photosynthetic system (35–37). As shown in Fig. 4A, the Fv/Fm values of the control group remained stable, ranging between 0.5 and 0.6. In contrast, the group treated with algicidal substance exhibited a decline in Fv/Fm values after 6 h, with the reduction becoming more pronounced over time. By the 12th hour, the Fv/Fm value had decreased by 50%, showing a highly significant difference compared to the control group. By the 24th hour, the Fv/Fm value had plummeted to 0.055, marking a 90.6% reduction. These results demonstrate that the algicidal substances severely impair the PSII pathway within 24 h of treatment.

PSII in oxygenic photosynthetic organisms primarily utilizes Chl-a as its major light-harvesting pigment (38), and Chl-a concentration serves as a key indicator of the damage to the photosynthetic system of cyanobacteria. A reduction in Chl-a levels reflects the disruption of light-harvesting capabilities and overall photosynthetic efficiency (39, 40). As shown in Fig. 4B, the Chl-a concentration in the control group remained stable at 1.8 mg/L. However, after treatment with 3 µg/mL of the algicidal substance, the Chl-a concentration in *L. fusiformis* decreased from 1.8 mg/L to 1.3 mg/L after 24 h, representing a 28% reduction. By 120 h, the Chl-a concentration further dropped to 0.24 mg/L, with an 86% reduction. Therefore, the algicidal substances inhibit the PSII pathway and degrade Chl-a in *L. fusiformis*, thereby disrupting the photosynthetic system.

### Screening and identification of strains inhibiting *H. variabilis* 2-9

**Fig 5:**
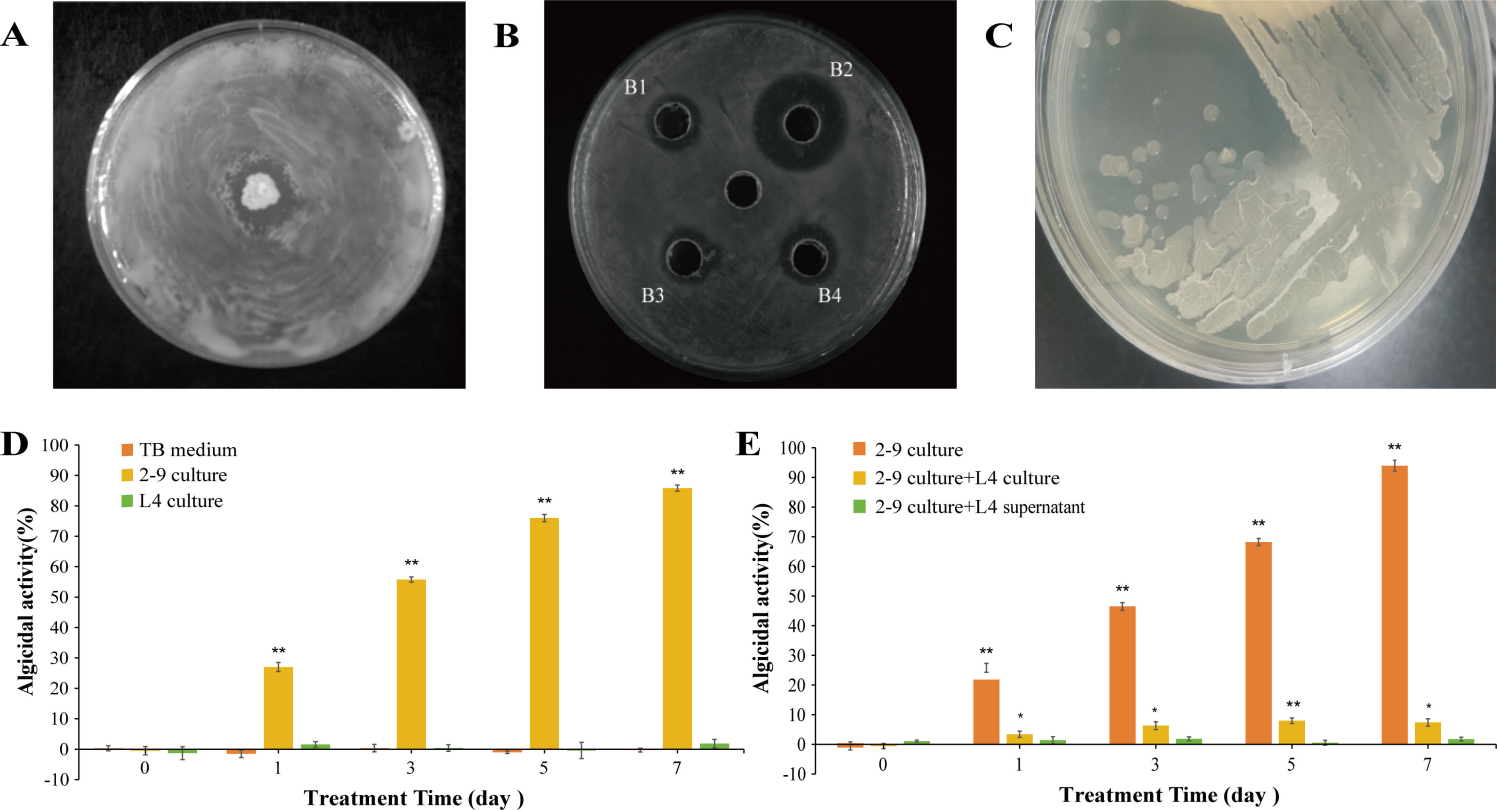
The isolation and prevention potential of *B. velezensis* L4 against *H. variabilis* 2-9. (**A**) *B. velezensis* L4 showing antimicrobial activity against *H. variabilis* 2-9. (**B**) Inhibitory effect of the cell-free supernatant of *B. velezensis* L4 against *H. variabilis* 2-9 when fermented in different medium: YT medium (B1), TB medium (B2), SOB medium (B3), SOC medium (B4). (**C**) Colonies of *B. velezensis* L4 growing on the TB agar medium. (**D**) Algicidal activity of *H. variabilis* 2-9 and *B. velezensis* L4 culture, with TB medium as control. (**E**) Algicidal activity of *H. variabilis* 2-9 alone and in combination with *B. velezensis* L4 culture or cell-free supernatant. All error bars indicate the standard error of three replicates. *, *P* < 0.05 **, *P* < 0.01 compared with the control group.

To counteract the negative impacts of *H. variabilis* 2-9, we screened 86 deep-sea sediment-derived bacterial strains for their inhibitory activity against this organism. Among these isolates, strain L4 and its cell-free supernatant (cultured in TB medium) exhibited the strongest antimicrobial activity against *H. variabilis* 2-9 (Fig. 5A and B). Further characterization of strain L4 revealed that it forms smooth, milky-white colonies on TB solid medium, which later develop wrinkled and rough textures (Fig. 5C). The 16S rRNA gene sequence of strain L4 was analyzed and showed high homology with *Bacillus velezensis* strain Hk9-21 (98.92%) and *Bacillus velezensis* strain SB2 (99.11%) in the NCBI database. Additionally, strain L4 is closely clustered with these reference strains in the phylogenetic tree (Fig. S3). Therefore, the deep-sea strain L4 was designated as *Bacillus velezensis* L4.

### Effect of *B. velezensis* L4 on the cultivation of *L. fusiformis*

To evaluate the biocontrol potential of *B. velezensis* L4 against *H. variabilis* 2-9 during *L. fusiformis* cultivation, we first detected the effect of *B. velezensis* L4 on *L. fusiformis*. As illustrated in Fig. 5D and B*. velezensis* L4 culture had no algicidal effect on *L. fusiformis*, while *H. variabilis* 2-9 culture exhibited obvious algicidal activity as early as the first day. These results indicate that *B. velezensis* L4 does not exhibit adverse effect on *L. fusiformis*, making it a promising candidate for biocontrol applications targeting *H. variabilis* 2-9.

Following confirmation that *B. velezensis* L4 exhibited a non-inhibitory effect on *L. fusiformis*, we evaluated its antagonistic activity against *H. variabilis* 2-9 in algal coculture systems. As shown in Fig. 5E, the algicidal activity of *H. variabilis* 2-9 culture was approximately 30% on the first day, gradually increasing over time. However, when co-cultured with either *B. velezensis* L4 or its cell-free supernatant, *H. variabilis* 2-9 exhibited significantly reduced algicidal activity. Additionally, we also observed the experimental phenomena under a microscope. The algal cells treated with *H. variabi*lis 2-9 appeared dissolved, while the algal cells co-cultured with *H. variabilis* 2-9 and *B. velezensis* L4 showed no significant differences compared to the control group (Fig. S4). Therefore, *B. velezensis* L4 can effectively mitigate the damage caused by *H. variabilis* 2-9, highlighting its potential to biocontrol *L. fusiformis* disease.

### Isolation and identification of active components of *B. velezensis* L4

**Fig 6:**
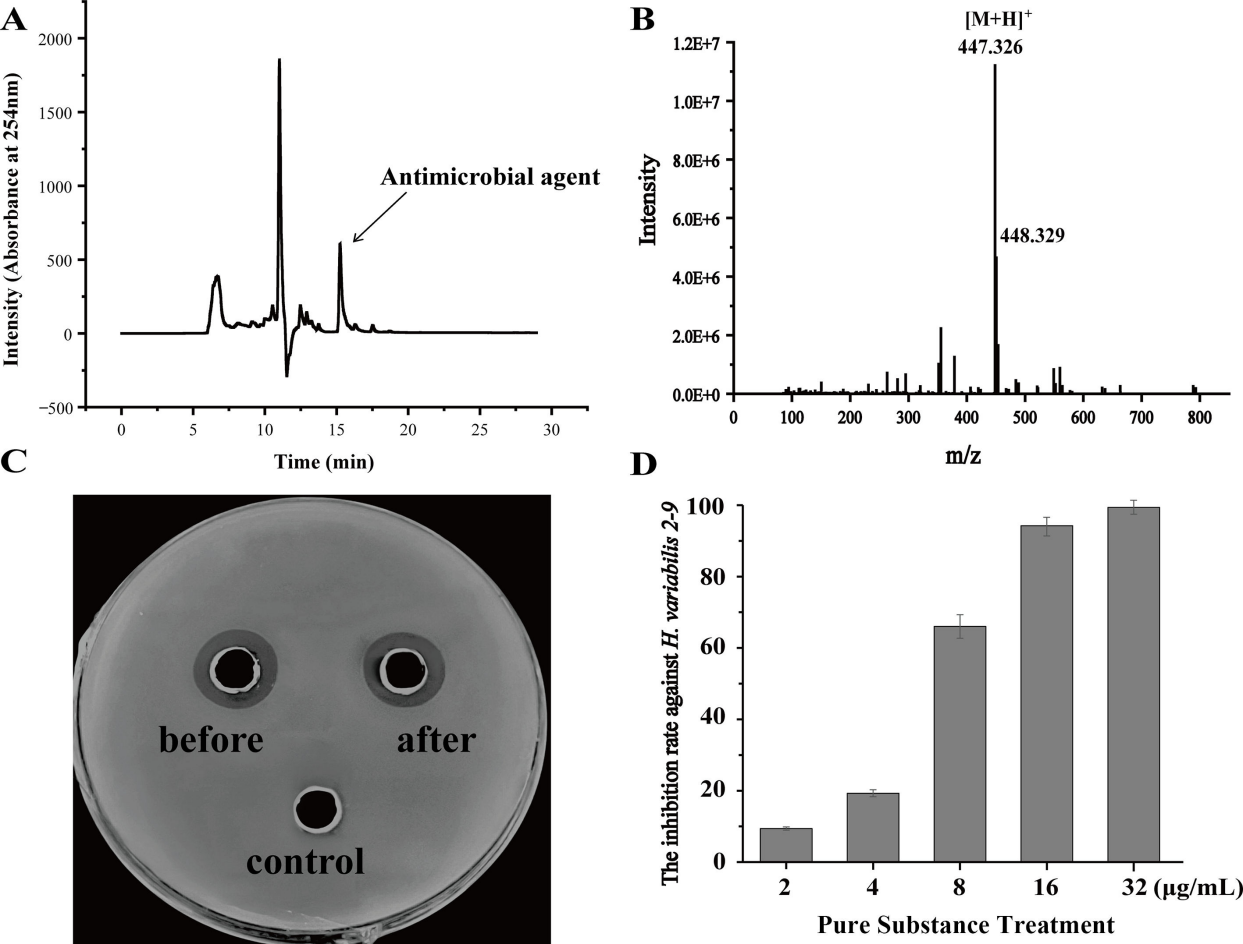
Determination of antimicrobial substance produced by *B. velezensis* L4. (**A**) RP-HPLC analysis of the antimicrobial substance produced by *B. velezensis* L4. (**B**) LC-MS analysis of the fraction eluted at 15.210 min. (**C**) The activity detection of the purified antibacterial substances against *H. variabilis* 2-9 before and after degradation, with methanol as the control. (**D**) Determination of the inhibition activity of the purified antimicrobial substances.

To isolate the potential antimicrobial agent, the supernatant of *B. velezensis* L4 was extracted with ethyl acetate, followed by methanol extraction, and the resulting crude extract was further purified using RP-HPLC. As shown in Fig. 6A, a fraction with a retention time of 15.210 min exhibited significant antimicrobial activity. The purified active fraction was further analyzed by LC-MS. As illustrated in Fig. 6B, a distinct peak was observed at an *m*/*z* value of 447.326, corresponding to the single protonated form [M + H]^+^ with a molecular formula of C_31_H_42_O_2_. However, during assessing the stability of the purified active substance, we observed that the purified active substance was highly susceptible to degradation even when we tried several methods to prevent the degradation (Fig. S5). Surprisingly, when we evaluated their antibacterial efficacy before and after degradation, we found no significant difference in their antimicrobial performance, indicating that this natural antimicrobial compound still retains substantial application potential even after degradation (Fig. 6C).

To evaluate the antibacterial activity of the purified active substance, we tested its efficacy against the pathogen *H. variabilis* 2-9 at concentrations of 2, 4, 8, 16, and 32 μg/mL. As shown in Fig. 6D, treatment of *H. variabilis* 2-9 with the purified substance at concentrations of 2 or 4 μg/mL resulted in a very low inhibition rate. However, when the concentration was increased to 8 μg/mL, the inhibition rate reached approximately 66.02%, and it rose to about 99.36% at 16 μg/mL, indicating that the purified substance exhibits potent antimicrobial activity.

## DISCUSSION

Previous studies have shown that *Halomonas* species produce a variety of secondary metabolites, which may exert algicidal effects by disrupting cell membrane integrity or interfering with photosynthetic pathways, and showed promising potential for controlling harmful algal blooms (41, 42). However, the precise chemical nature of the algicidal compounds and their molecular targets remain unclear. In this study, the 48-h fermented supernatant of *H. variabilis* 2-9 exhibited potent algicidal activity against *L. fusiformis* and other cyanobacterial species. Notably, the primary algicidal compounds were identified as dibutyl phthalate (DBP), a substance known to be both chemically synthesized and naturally produced by plants, fungi, and bacteria (43). However, DBP is classified as a highly hazardous compound, posing significant risks to human health, aquatic organisms, and ecosystems (44–46). Consequently, the application of *Halomonas* for harmful algal blooms requires careful risk-benefit evaluation due to these biosafety. Moreover, in *L. fusiformis* aquaculture systems, *Halomonas* contamination not only causes economic losses but also raises serious environmental and health risks. Thus, developing targeted strategies to prevent and manage *Halomonas* contamination represents an urgent priority for sustainable aquaculture and ecosystem protection.

Bacterial-algal interactions involve complex direct and indirect mechanisms (47). While direct interactions require physical contact, indirect interactions are mediated by secreted compounds that disrupt algal physiological processes, including photosynthesis and antioxidant systems, ultimately leading to cellular damage (36, 37, 48). Our study demonstrates that *H. variabilis* 2-9 exerts its algicidal effect primarily through indirect means by secreting bioactive compounds that target the photosynthetic apparatus of *L. fusiformis*. Notably, we observed a rapid decline in the Fv/Fm ratio within 24 h of treatment, while chlorophyll-a concentrations showed delayed changes. This temporal pattern indicates that impairment of photosystem II efficiency, as reflected by the Fv/Fm ratio, represents a primary algicidal mechanism of *H. variabilis* 2-9 against *L. fusiformis*.

The Fv/Fm ratio is a direct indicator of the maximum photochemical efficiency of the PSII reaction center. Under adverse conditions, the protein complex within the PSII reaction center is typically the first to be damaged, resulting in a rapid decline in the Fv/Fm ratio (49). Chl-a is a vital component of photosynthetic pigments, present not only in the PSII reaction center but also in the antenna pigment complex. Chl-a degradation only takes place when stress intensifies and the damage extends to the antenna pigment system (50). Additionally, spirulina cells possess inherent pigment repair mechanisms that can delay chlorophyll a degradation during the early stages of stress (51). As a result, the decrease in Chl-a content occurs later than the changes observed in the Fv/Fm ratio, which is consistent with our observation.

*Bacillus velezensis* has been widely documented as a versatile bacterium with rapid growth characteristics and the ability to produce diverse secondary metabolites, including antimicrobial proteins and lipopeptide antibiotics (52). Recognized as an eco-friendly alternative to synthetic fungicides, this species demonstrates dual functionality as both a plant growth-promoting rhizobacterium and a biocontrol agent, effectively suppressing crop pathogens while enhancing agricultural yields (53, 54). Furthermore, it has been used as a probiotic or feed additive in fish and poultry diets (55, 56). Given these properties, *B. velezensis* holds significant potential for multifunctional applications in agriculture and food industries. In this study, strain *B. velezensis* L4 was found to produce an antimicrobial agent that effectively suppressed the pathogen *H. variabilis* 2-9 without damaging *L. fusiformis*, highlighting its promising potential as a biocontrol agent for managing cyanobacterial diseases.

## MATERIALS AND METHODS

### Algal species and culture condition

*Limnospira fusiformis* (provided by Qingdao Institute of Bioenergy and Process, Chinese Academy of Sciences) was cultured in Zarrouk medium as described in a previous report (57). Other algae used in the experiment were *Anabaena* sp. FACHB-1140 and FACHB-188, *Dolichospermum* sp. FACHB-1250, *Dolichospermum flos-aquae* FACHB-1255, were purchased from the Freshwater Algae Species Bank at the Chinese Academy of Sciences. These algae were cultivated in BG11 culture medium at 25 ± 0.5°C with a 12-h light/dark cycle and 1,000 lux of light intensity. Algae cultures were manually shaken every 12 h, and exponential cells with an initial density of 5 × 10^6^ cells/mL were used in this study, as previously reported (37).

### Isolation and identification of *L. fusiformis* pathogen

To quickly screen the pathogen, the healthy and decayed *L. fusiformis* samples were first subjected to 16S rRNA gene profiling. To isolate the pathogen, the supernatant of the diseased *L. fusiformis* culture was serially diluted, and 100 µL of each diluted sample was evenly spread onto LB, 2216E, or BG11 agar medium, and incubated at 28°C until distinct colonies emerged. The individual colonies were then inoculated into broth medium and incubated at 28°C for 48 h. Subsequently, 2% (vol/vol) of bacterial cultures or their supernatants (obtained by centrifugation at 8,000 rpm for 10 min and filtration through a 0.22 µm membrane) was transferred into 12-well plate containing healthy *L. fusiformis* cultures. The plates were incubated at 25 ± 0.5°C under a light intensity of 1,000 lux with a 12-h light/dark cycle, and the growth of *L. fusiformis* was monitored daily. A blank control group (no treatment) and a negative control group (supplemented with broth medium) were included. All experiments were performed in triplicate.

To identify the isolated pathogen, the 16S rRNA gene of the strain was amplified by PCR using the universal primers 27F and 1492R. The resulting sequences were then analyzed using BLAST against the National Center for Biotechnology Information (NCBI) database for identification. Additionally, cellular morphology was examined via transmission electron microscopy (TEM) for further characterization.

### Algicidal activity test

Although the pathogenic strain 2-9 was isolated from diseased algae cultured in Zarrouk medium, it exhibited slow growth when grown exclusively in this medium. The 2216E medium provides a slightly alkaline environment that supports the normal growth of the pathogenic strain 2-9 and algae. Therefore, we chose to use 2216E medium for our subsequent experiments to ensure normal growth rates. To evaluate the algicidal activity of the pathogen at different fermentation times, an overnight culture of the pathogenic strain was inoculated into 2216E broth medium at a concentration of 1% (vol/vol) and incubated at 28°C with shaking at 150 rpm for 24, 48, and 72 h, respectively. After fermentation, the cultures were centrifuged at 8,000 rpm for 20 min to obtain cell-free supernatants. These supernatants were then added to 12-well plates containing 3 mL of *L. fusiformis* culture in the exponential growth phase at a concentration of 2% (vol/vol). The plates were incubated at 25 ± 0.5°C under a light intensity of 1,000 lux with a 12-h light/dark cycle. A control group was established by adding 2% (vol/vol) broth medium. Each experimental condition was performed in triplicate, and algicidal activity was monitored and recorded at 0, 1, 3, 5, and 7 days.

To assess the algicidal spectrum of the pathogen, other algal strains, such as *Anabaena* sp. FACHB-1140 and FACHB-188, *Dolichospermum* sp. FACHB-1250, *Dolichospermum flos-aquae* FACHB-1255, were employed as test organisms. The cell-free supernatant of the pathogen was added to 12-well plates containing 3 mL of algal culture in the exponential growth phase at a concentration of 2% (vol/vol). A control group was established by adding 2% (vol/vol) broth medium. Each experimental condition was conducted in triplicate, and algicidal activity was monitored and documented at 0, 1, 3, 5, and 7 days.

The algicidal activity was measured by chlorophyll-a autofluorescence, with an excitation wavelength of 440 nm and an emission wavelength of 680 nm, as previously described (36). Algicidal activity was calculated using the formula: algicidal activity % = (1 − *F_t_ / F_0_*) ×100%, where *F_t_* and *F_0_* represent the fluorescence values of the algal cultures at different time points and at the beginning of the treatment period, respectively.

### Isolation and identification of algicidal substances

To identify the algicidal compounds, the selected bacterial strain was cultured in broth medium for 48 h at 28°C. The cell-free supernatant was then extracted with an equal volume of ethyl acetate, followed by thorough mixing until phase separation occurred. The supernatant was transferred to a round-bottom flask and evaporated at 50°C until the ethyl acetate was completely removed. The residual solid was subsequently redissolved in methanol to yield the crude extract. For further purification, the crude extract was subjected to reversed-phase high-performance liquid chromatography (RP-HPLC) using an Agilent system equipped with an Eclipse XDB-C18 column (5 µm, 9.4 × 250 mm). The column was eluted at a flow rate of 2 mL/min under the following gradient conditions: 0–5 min, mobile phase B increased from 0% to 85%; 5–20 min, mobile phase B increased from 85% to 100%; 20–40 min, mobile phase B was maintained at 100%. Mobile phase A consists of 70% (vol/vol) methanol, while mobile phase B is composed of 100% methanol. Elution fractions were collected for subsequent analysis.

To preliminarily identify the purified algicidal substance, liquid chromatography coupled with mass spectrometry (LC-MS) was employed according to previously reported (58). A linear ion trap Orbitrap spectrometer (Thermo Fisher, USA) equipped with high-energy collision-induced dissociation (HCD) was used. The spray voltage was set to 3 kV, and the ion transfer capillary temperature was maintained at 275 °C. To further confirm the principal algicidal substance, commercially available pure compounds were obtained, and their liquid chromatography profiles and algicidal activities were compared to that of the algicidal substances.

To quantitatively assess the algicidal efficacy of the active compounds, the algal culture in the exponential growth phase was exposed to the substance at final concentrations of 1, 2, 3, 4, 5, and 6 µg/mL dissolved in methanol. A control group was treated with methanol alone. Each experiment was performed in triplicate, and the algicidal activity was monitored and recorded at 0, 1, 3, 5, and 7 days.

### Effect of the algicidal substances on photosynthesis in *L. fusiformis*

To further investigate the physiological responses of *L. fusiformis* to algicidal compounds, we evaluated its Fv/Fm of photosystem II (PS II) and the chlorophyll-a (Chl-a) concentration. *L. fusiformis* cultures treated with 3 µg/mL of the algicidal substance for 0, 6, 12, 18, and 24 h were used for Fv/Fm measurements, with methanol-treated cultures serving as the control. Notably, Fv/Fm measurements were conducted after a 15-min dark adaptation period to ensure accurate assessment of photosynthetic efficiency. For chlorophyll-a determination, *L. fusiformis* cultures treated with 3 µg/mL of the algicidal substance for 0, 24, 48, 72, 96, and 120 h were used for Chl-a measurement. Absorbance of the supernatant was measured at 663 nm and 645 nm using a spectrophotometer, with ethanol serving as the blank. The Chl-a content was calculated following the formula: Chl-a = 84.60A_663_ − 83.89A_645_ as described previously (59).

### Screening and identification of strains inhibiting *L. fusiformis* pathogen

To screen bacteria capable of biological controlling the cyanobacterial contamination, deep-sea sediments were subjected to serial dilution using sterile seawater. Then, 100 µL of each diluted sample was evenly spread onto 2216E agar medium and incubated at 28°C. Colonies exhibiting distinct morphological characteristics, such as variations in shape, size, and pigmentation, were selectively isolated and purified for further analysis. The agar well diffusion method was used to screen bacteria inhibiting the growth of *L. fusiformis* pathogen following the previous method (60). Specifically, an overnight culture of the pathogen was mixed with 2216E medium and poured into plates. Wells were then created in the agar, and 100 µL of the cell-free supernatant from the isolated strain was added to the wells. The plates were incubated overnight, and strains exhibiting clear inhibition zones around the wells were identified as potential antimicrobial strains against *L. fusiformis* pathogen. For the identification of antimicrobial strains, the 16S rRNA gene was amplified using universal primers 27F and 1492R, and the resulting sequences were analyzed using the BLAST tool in NCBI database.

### Assessment of the effects of potential antimicrobial strains on the cultivation of *L. fusiformis*

To ensure that potential antimicrobial strains do not adversely affect *L. fusiformis* while controlling pathogenic infections, their impact on the algae was evaluated. Briefly, overnight cultures of the pathogenic strain and the potential antimicrobial strains were introduced into 12-well plates at a concentration of 2% (vol/vol), respectively. Each well contained 3 mL of *L. fusiformis* culture in the exponential growth phase. The plates were incubated at 25 ± 0.5°C under a light intensity of 1,000 lux with a 12-h light/dark cycle. The wells with the addition of corresponding medium at the concentration of 2% (vol/vol) were used as blank controls. Each experimental condition was performed in triplicate, and algicidal activity was monitored and recorded at 0, 1, 3, 5, and 7 days to assess the compatibility of the antimicrobial strains with *L. fusiformis*.

To evaluate the efficacy of antimicrobial strains in mitigating the detrimental effects of the pathogen on *L. fusiformis* within a cyanobacterial culture system, a combined treatment approach was employed. Initially, an overnight culture of pathogenic bacteria was introduced at a concentration of 2% (vol/vol) into 12-well plates containing 3 mL of *L. fusiformis* culture in the exponential growth phase as a pretreatment. Subsequently, either the bacterial culture or the cell-free supernatant (obtained by centrifugation at 8,000 rpm for 10 min and filtration through a 0.22 µm membrane) of the antimicrobial strains was added at a concentration of 2% (vol/vol) as the treatment. Wells with the addition of 2% (vol/vol) corresponding medium were used as a blank control, and the pretreatment group was set as a reference. Each experiment was conducted in triplicate, and algicidal activity was monitored and recorded at 0, 1, 3, 5, and 7 days to assess the protective effects of the antimicrobial strains against pathogenic bacterial damage.

### Identification and evaluation of the antimicrobial compounds

To isolate the active compounds, the antimicrobial strains were cultured in TB liquid medium at an initial concentration of 1% (vol/vol) and fermented at 28°C with shaking at 150 rpm for 48 h. The cell-free supernatant was obtained by centrifuging the culture at 8,000 rpm for 20 min. The crude extract was then prepared by liquid-liquid extraction using an equal volume of ethyl acetate. For further purification, the crude extract was subjected to RP-HPLC using an Agilent system equipped with an Eclipse XDB-C18 column (5 µm, 9.4 × 250 mm). The column was eluted at a flow rate of 2 mL/min under the following gradient conditions: 0–5 min, mobile phase B increased from 0% to 90%; 5–30 min, mobile phase B increased from 90% to 100%; 30–40 min, mobile phase B was maintained at 100%. Mobile phase A consisted of 70% (vol/vol) methanol, while mobile phase B is 100% methanol. Elution fractions were collected. The purified fractions were subsequently analyzed and identified using LC-MS.

To evaluate the antimicrobial activity of the purified active substances, the active fraction from RP-HPLC was collected and concentrated, and then the antimicrobial activity against pathogen *H. variabilis* 2-9 was detected. Briefly, an overnight culture of *H. variabilis* 2-9 was inoculated into 2216E liquid medium at a ratio of 0.5% (vol/vol). The mixture was then distributed into a 96-well cell culture plate, with 180 μL per well. Subsequently, 20 μL of the purified active substances was added to each well, achieving final concentrations of 2, 4, 8, and 16 μg/mL, respectively. An equal volume of methanol was added to control wells. The 96-well plate was incubated at 28°C on a rotary shaker at 150 rpm for 48 h, and cell growth was measured at 600 nm using a microplate reader (Infinite M1000 Pro; Tecan, Mannedorf, Switzerland). The growth inhibition rate was calculated by normalizing the growth of *H. variabilis* 2-9 treated with the purified substances to that of the control group treated with an equal volume of methanol. Each treatment was performed in triplicate.

## Data Availability

The 16S rRNA genomic sequence of *H. variabilis* 2-9 has been deposited in the NCBI database with GenBank accession number PV367401. The 16S rRNA genomic sequence of *Bacillus velezensis* L4 has been deposited in the NCBI database with GenBank accession number PV367488.

## SUPPLEMENTAL MATERIAL

Supplemental material aem.00199-26-s0001.docx Fig. S1 to S5; Table S1.

ASM does not own the copyrights to Supplemental Material that may be linked to, or accessed through, an article. The authors have granted ASM a non-exclusive, world-wide license to publish the Supplemental Material files. Please contact the corresponding author directly for reuse.
