# Supplementary material for: Pathogenic effects of Halomonas on cyanobacteria and biocontrol potential of a deep-sea Bacillus strain
Source: Appl Environ Microbiol. 2026 Mar 20;92(4):e00199-26. doi: 10.1128/aem.00199-26 (PMC13101475; doi:10.1128/aem.00199-26)
Supplement: Supplemental material — Fig. S1 to S5; Table S1. [file aem.00199-26-s0001.docx]

Supporting Information for

**Pathogenic effects of *Halomonas* on cyanobacteria and biocontrol potential of a deep-sea *Bacillus* strain**

Xinyi Li^1^*, MeilinYuan^1^* Chaomin Sun^2,3^ Shimei Wu^1^**

^1^ College of Life Sciences, Qingdao University, Qingdao, 266071, China.

^2^ CAS Key Laboratory of Experimental Marine Biology, Institute of Oceanology, Chinese Academy of Sciences, Qingdao, 266071, China

^3^ Laboratory for Marine Biology and Biotechnology, Qingdao National Laboratory for Marine Science and Technology, Qingdao, 266071, China

^*^ Xinyi Li and MeilinYuan contributed equally to this article.

^**^ Corresponding author

Shimei Wu E-mail: shimeiwu2016@126.com

**KEYWORDS:** Cyanobacteria, *Limnospira fusiformis*, *Halomonas*, pathogen, biocontrol

**Running title:** Identification and biocontrol of cyanobacteria pathogen


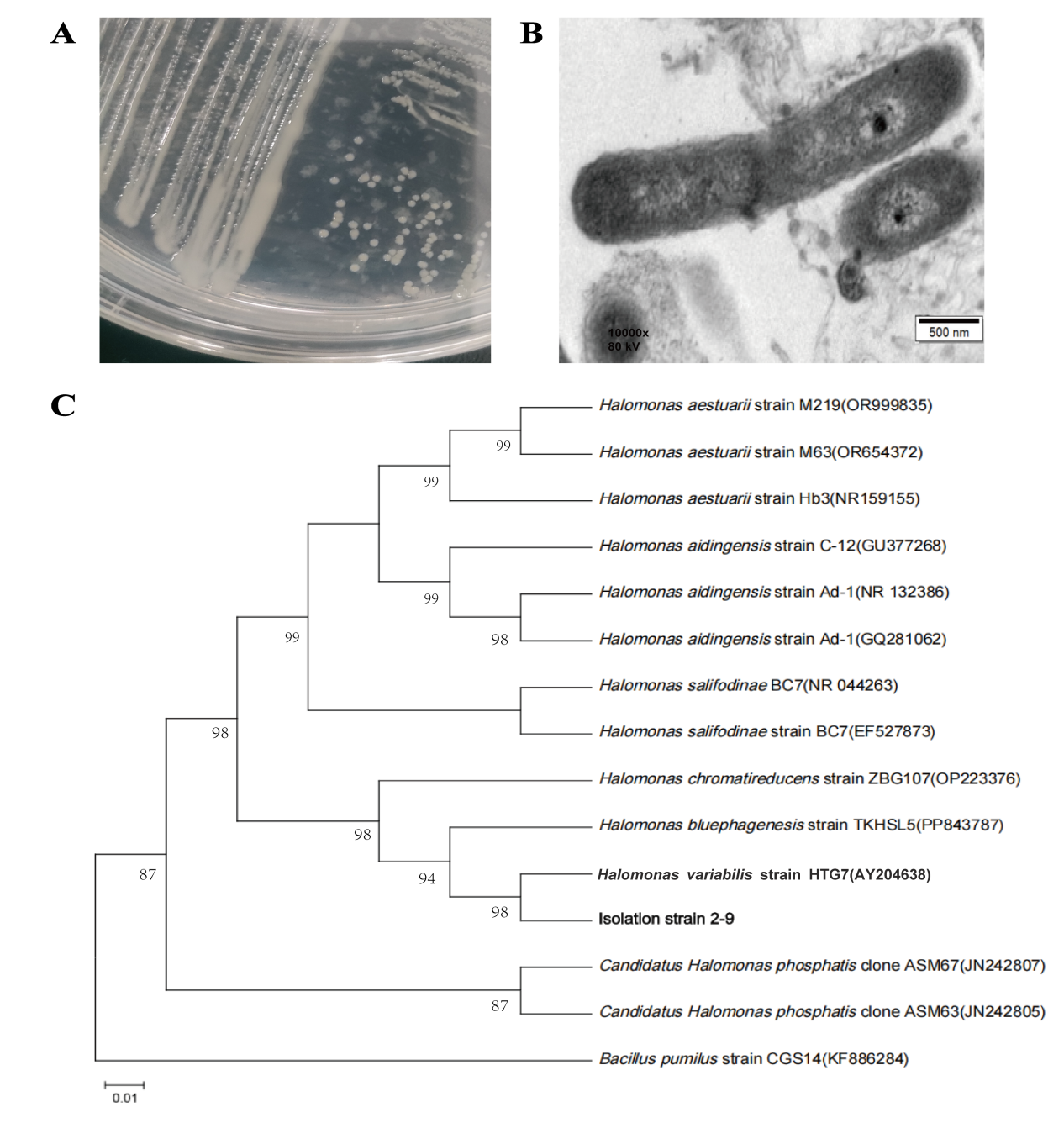


Figure S1 Phylogenetic tree based on 16S rRNA gene sequence of strain 2-9.

**
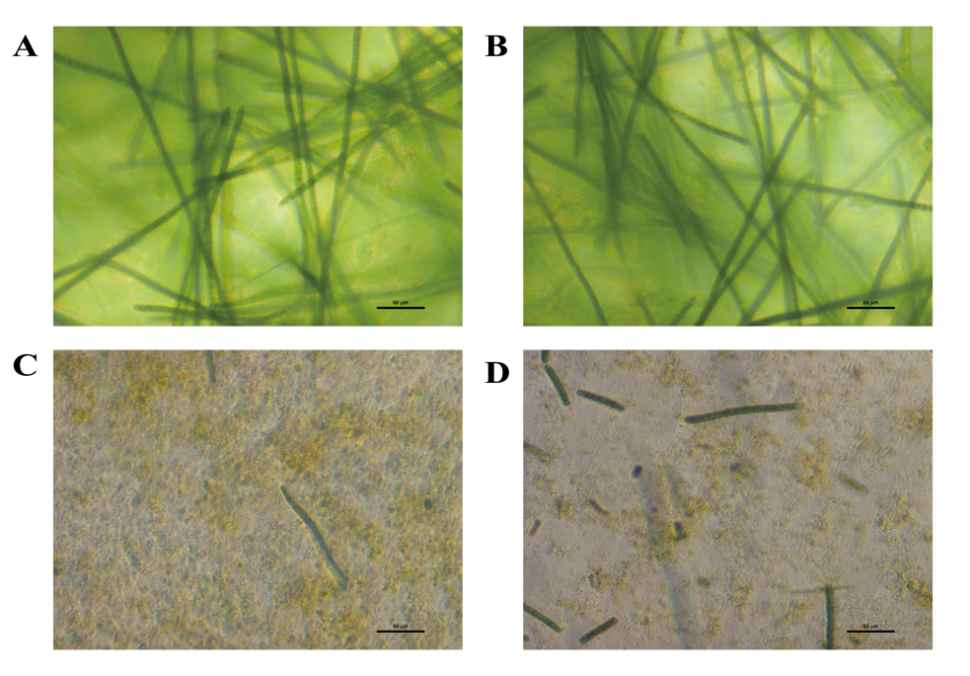
**

Figure S2 The morphology of algae observed under the microscope as follows: A represents the algae without any treatment. B shows the control group treated with 2216E medium, C is algae treated with *H. variabilis* 2-9 culture, and D illustrates algae treated with *H. variabilis* 2-9 supernatant


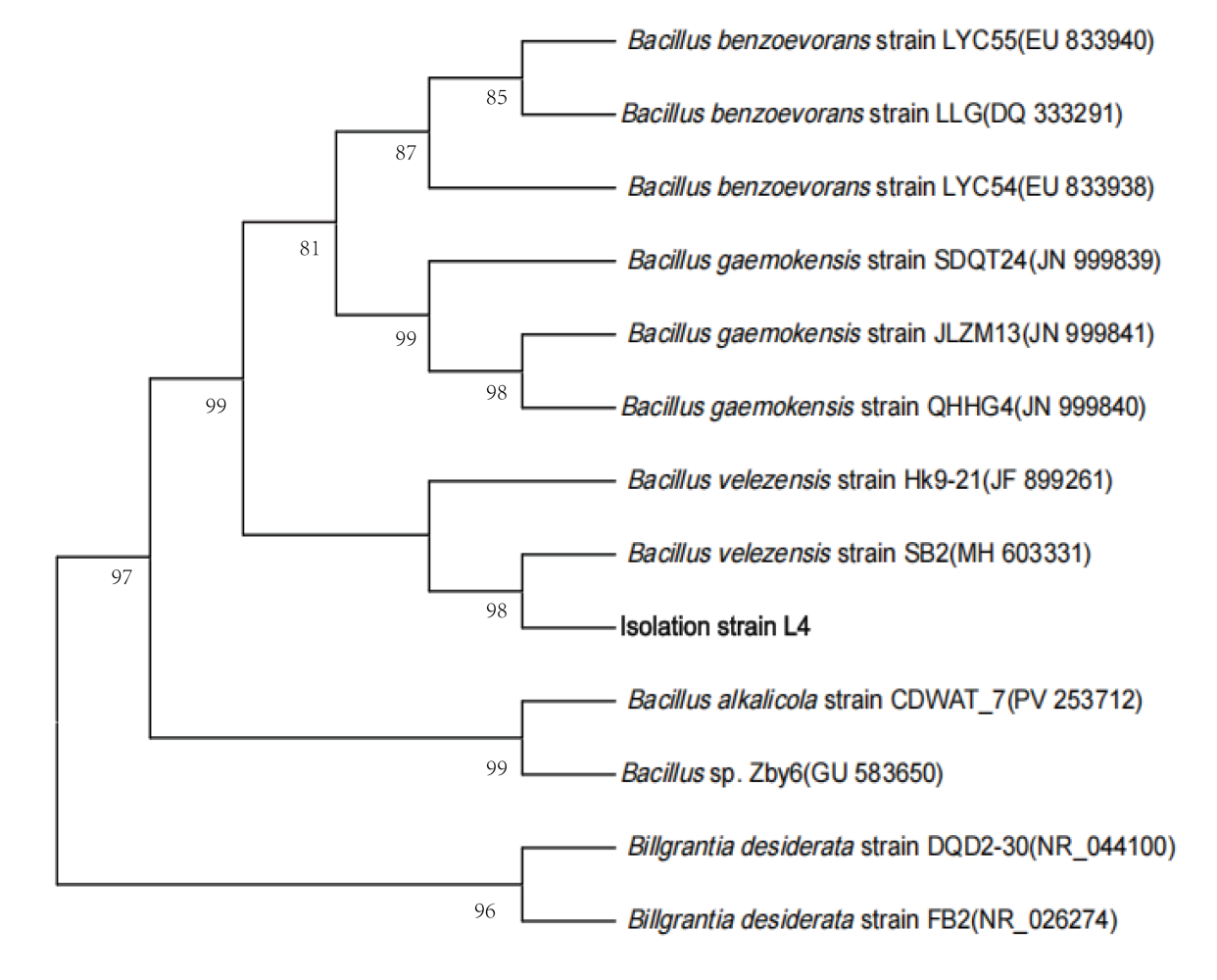


Figure S3 Phylogenetic tree based on 16S rRNA gene sequence of strain L4.


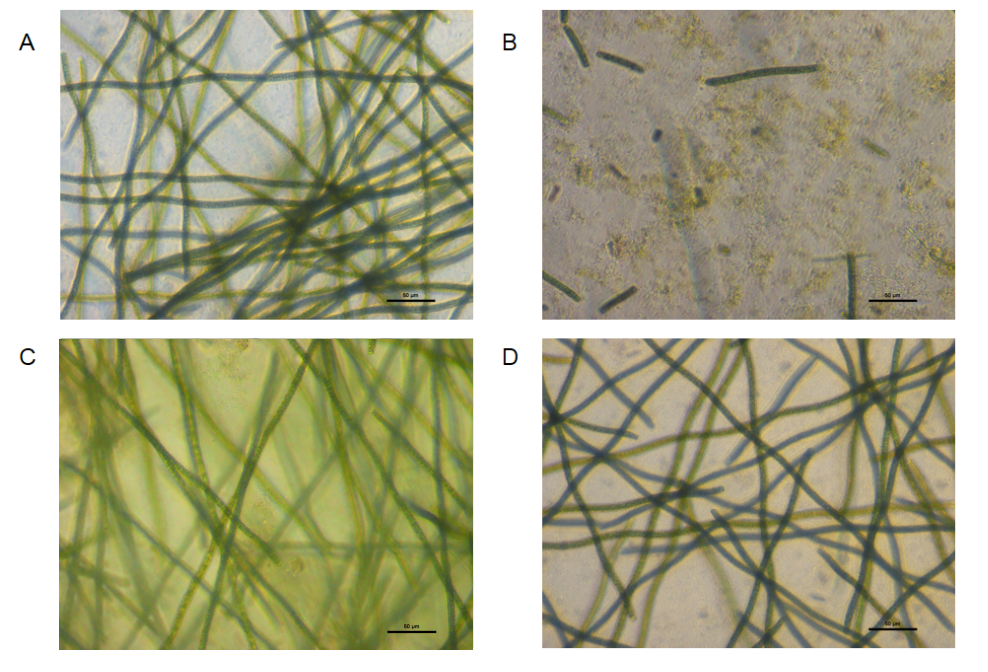


Figure S4 The morphology of algae observed under the microscope as follows: A represents the control group treated with TB medium, B shows algae treated with *H. variabilis* 2-9 culture, C presents algae treated with *B. velezensis* L4 culture, and D illustrates algae treated with a combination of *H. variabilis* 2-9 culture and *B. velezensis* L4 culture.


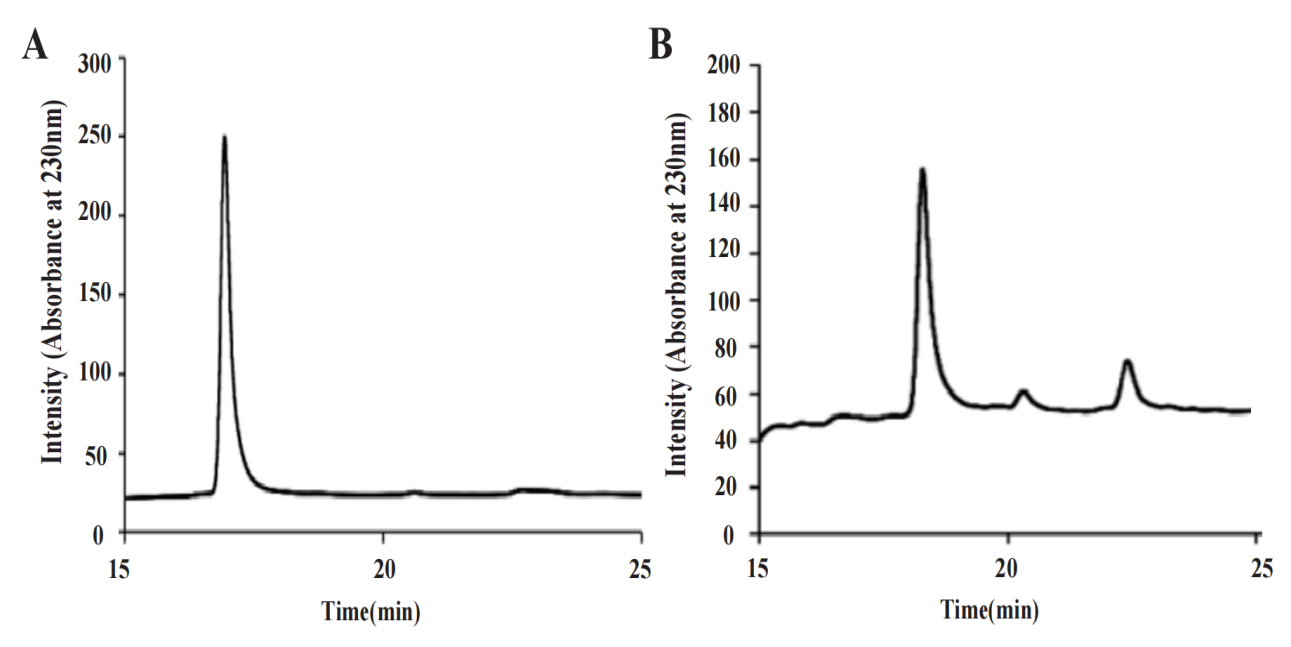


Figure S5 The reversed-phase liquid chromatogram of the purified active substance produced by *B. velezensis* L4: A illustrates the liquid phase diagram of the active substance detected immediately after purification, while B presents the liquid phase diagram of the active substance analyzed two days later after purification.

**Table S1 The identification of the other ten *Halomonas* strains based on 16S rRNA**

| Number | species | Pre. Ident | 16S rRNA |
| --- | --- | --- | --- |
| 2-1 | *Halomonas qaidamensis* | 98.04% | CTGGGCGCGGCTACACATGCAAGTCGAGCGGTAACAGGGGTAGCTTGCTACCCGCTGACGAGCGGCGGACGGGTGAGTAATGCATAGGAATCTGCCCGGTAGTGGGGGATAACCTGGGGAAACCCAGGCTAATACCGCATACGTCCTACGGGAGAAAGGGGGCTTCGGCTCCCGCTATTGGATGAGCCTATGTCGGATTAGCTAGTTGGTGAGGTAATGGCTCACCAAGGCGACGATCCGTAGCTGGTCTGAGAGGATGATCAGCCACATCGGGACTGAGACACGGCCCGAACTCCTACGGGAGGCAGCAGTGGGGAATATTGGACAATGGGCGGAAGCCTGATCCAGCCATGCCGCGTGTGTGAAGAAGGCCTTCGGGTTGTAAAGCACTTTCAGCGAGGAAGAACGCCTAGTGGTTAATACCCATTAGGAAAGACATCACTCGCAGAAGAAGCACCGGCTAACTCCGTGCCAGCAGCCGCGGTAATACGGAGGGTGCGAGCGTTAATCGGAATTACTGGGCGTAAAGCGCGCGTAGGTGGCTTGATAAGCCGGTTGTGAAAGCCCCGGGCTCAACCTGGGAACGGCATCCGGAACTGTCAAGCTAGAGTGCAGGAGAGGAAGGTAGAATTCCCGGTGTAGCGGTGAAATGCGTAGAGATCGGGAGGAATACCAGTGGCGAAGGCGGCCTTCTGGACTGACACTGACACTGAGGTGCGAAAGCGTGGGTAGCAAACAGGATTAGATACCCTGGTAGTCCACGCCGTAAACGATGTCGACCAGCCGTTGGGTGCCTAGCGCACTTTGTGGGGGAAGTTAACGCGATAAATCCACCGCCTGGGGGAGAACCGCCCGCAAGGTTAAACTCCAATGGA |
| 2-2 | *Halomonas qaidamensis* | 96.58% | TGGGGGTCGGACTACCATGCAAGTCGAGCGGTAACAGGGGTAGCTTGCTACCCGCTGACGAGCGGCGGACGGGTGAGTAATGCATAGGAATCTGCCCGGTAGTGGGGGATAACCTGGGGAAACCCAGGCTAATACCGCATACGTCCTACGGGAGAAAGGGGGCTTCGGCTCCCGCTATTGGATGAGCCTATGTCGGATTAGCTAGTTGGTGAGGTAATGGCTCACCAAGGCGACGATCCGTAGCTGGTCTGAGAGGATGATCAGCCACATCGGGACTGAGACACGGCCCGAACTCCTACGGGAGGCAGCAGTGGGGAATATTGGACAATGGGCGGAAGCCTGATCCAGCCATGCCGCGTGTGTGAAGAAGGCCTTCGGGTTGTAAAGCACTTTCAGCGAGGAAGAACGCCTAGTGGTTAATACCCATTAGGAAAGACATCACTCGCAGAAGAAGCACCGGCTAACTCCGTGCCAGCAGCCGCGGTAATACGGAGGGTGCGAGCGTTAATCGGAATTACTGGGCGTAAAGCGCGCGTAGGTGGCTTGATAAGCCGGTTGTGAAAGCCCCGGGCTCAACCTGGGAACGGCATCCGGAACTGTCAAGCTAGAGTGCAGGAGAGGAAGGTAGAATTCCCGGTGTAGCGGTGAAATGCGTAGAGATCGGGAGGAATACCAGTGGCGAAGGCGGCCTTCTGGACTGACACTGACACTGAGGTGCGAAAGCGTGGGTAGCAAACAGGATTAGATACCCTGGTAGTCCACGCCGTAAACGATGTCGACCACCCCGTTGGGTGCCTAACGCACTTTGTTGGGGAAGTTAACGCGGTAAATCCACCGCCCGGGGGAGAACCGCCGCCAGGGTAAAACTCCAATGAATTGTACGGGGGCCCCCCCAACCGGGGGGAAATGTGTGTTTAT |
| 2-3 | *Halomonas nitrilica* | 96.54% | CTGACGTAGGCTACACATGCAAGTCGAGCGGCAGCACGGGAAGCTTGCTTCCTGGTGGCGAGCGGCGGACGGGTGAGTAATGCATAGGAATCTGCCCGGTAGTGGGGGATAACCTGGGGAAACCCAGGCTAATACCGCATACGCCCTACGGGGGAAAGCAGGGGCTCTTCGGACCTTGCGCTATCGGATGAGCCTATGTCGGATTAGCTGGTTGGTGAGGTAATGGCTCACCAAGGCGACGATCCGTAGCTGGTCTGAGAGGATGATCAGCCACATCGGGACTGAGACACGGCCCGAACTCCTACGGGAGGCAGCAGTGGGGAATATTGGACAATGGGCGCAAGCCTGATCCAGCCATGCCGCGTGTGTGAAGAAGGCCCTCGGGTTGTAAAGCACTTTCAGTGGGGAAGAAAGCCTGGTGGTTAATACCCGTCAGGAAGGACATCACCCACAGAAGAAGCACCGGCTAACTCCGTGCCAGCAGCCGCGGTAATACGGAGGGTGCGAGCGTTAATCGGAATTACTGGGCGTAAAGCGCGCGTAGGTGGCTTGATAAGCCGGTTGTGAAAGCCCCGGGCTCAACCTGGGAACGGCATCCGGAACTGTCAGGCTAGAGTGCAGGAGAGGAAGGTAGAATTCCCGGTGTAGCGGTGAAATGCGTAGAGATCGGGAGGAATACCAGTGGCGAAGGCGGCCTTCTGGACTGACACTGACACTGAGGTGCGAAAGCGTGGGTAGCAAACAGGATTAGATACCCTGGTAGTCCACGCCGTAAACGATGTCGACTAGCCGTTGGGTCCTTCGCGGACTTTGTGGCGCAGTTAACGCGATAAGTCGACCGCCTGGGGAGTACGGCCGCAAGGTTAAACTCAAATGAATTGACGGGGGCCCGCACAAGGGGGGGAGGATGTGGGTTTATTCCATGGACCG |
| 2-4 | *Halomonas qaidamensis* | 95.65% | CCGGGCGTCGGCTACCATGCAAGTCGAGCGGTAACAGGGGTAGCTTGCTACCCGCTGACGAGCGGCGGACGGGTGAGTAATGCATAGGAATCTGCCCGGTAGTGGGGGATAACCTGGGGAAACCCAGGCTAATACCGCATACGTCCTACGGGAGAAAGGGGGCTTCGGCTCCCGCTATTGGATGAGCCTATGTCGGATTAGCTAGTTGGTGAGGTAATGGCTCACCAAGGCGACGATCCGTAGCTGGTCTGAGAGGATGATCAGCCACATCGGGACTGAGACACGGCCCGAACTCCTACGGGAGGCAGCAGTGGGGAATATTGGACAATGGGCGGAAGCCTGATCCAGCCATGCCGCGTGTGTGAAGAAGGCCTTCGGGTTGTAAAGCACTTTCAGCGAGGAAGAACGCCTAGTGGTTAATACCCATTAGGAAAGACATCACTCGCAGAAGAAGCACCGGCTAACTCCGTGCCAGCAGCCGCGGTAATACGGAGGGTGCGAGCGTTAATCGGAATTACTGGGCGTAAAGCGCGCGTAGGTGGCTTGATAAGCCGGTTGTGAAAGCCCCGGGCTCAACCTGGGAACGGCATCCGGAACTGTCAAGCTAGAGTGCAGGAGAGGAAGGTAGAATTCCCGGTGTAGCGGTGAAATGCGTAGAGATCGGGAGGAATACCAGTGGCGAAGGCGGCCTTCTGGACTGACACTGACACTGAGGTGCGAAAGCGTGGGTAGCAAACAGGATTAGATACCCTGGTAGTCCACGCCGTAAACGATGTCGACCAGCCCGTTGGGTGCCTAGCGCACTTTGGGGGCGAAGTTAACGCGATAAGTCGACCGCCCGGGGGAAGACCGGCCGCAAGGGTAAACTCCAAGGAGTTGTGGGGGGGCCCCCCAAAGGGGGGAGGAGTGTGGGTTATTTCTTGTGGCCGCGGAGAACCTTTCCTATCTTTGGATCT |
| 2-5 | *Halomonas qaidamensis* | 99.15% | CGGCGTCGGTCTACACATGCAAGTCGAGCGGTAACAGGGGTAGCTTGCTACCCGCTGACGAGCGGCGGACGGGTGAGTAATGCATAGGAATCTGCCCGGTAGTGGGGGATAACCTGGGGAAACCCAGGCTAATACCGCATACGTCCTACGGGAGAAAGGGGGCTTCGGCTCCCGCTATTGGATGAGCCTATGTCGGATTAGCTAGTTGGTGAGGTAATGGCTCACCAAGGCGACGATCCGTAGCTGGTCTGAGAGGATGATCAGCCACATCGGGACTGAGACACGGCCCGAACTCCTACGGGAGGCAGCAGTGGGGAATATTGGACAATGGGCGGAAGCCTGATCCAGCCATGCCGCGTGTGTGAAGAAGGCCTTCGGGTTGTAAAGCACTTTCAGCGAGGAAGAACGCCTAGTGGTTAATACCCATTAGGAAAGACATCACTCGCAGAAGAAGCACCGGCTAACTCCGTGCCAGCAGCCGCGGTAATACGGAGGGTGCGAGCGTTAATCGGAATTACTGGGCGTAAAGCGCGCGTAGGTGGCTTGATAAGCCGGTTGTGAAAGCCCCGGGCTCAACCTGGGAACGGCATCCGGAACTGTCAAGCTAGAGTGCAGGAGAGGAAGGTAGAATTCCCGGTGTAGCGGTGAAATGCGTAGAGATCGGGAGGAATACCAGTGGCGAAGGCGGCCTTCTGGACTGACACTGACACTGAGGTGCGAAAGCGTGGGTAGCAAACAGGATTAGATACCCTGGTAGTCCACGCCGTAAACGATGTCGACCAGCCGTTGGGTGCCTAGCGCACTTTGTGGCGAAGTTAACGCGATAAGTCGACCGCCTGGGGAGTACGGCCGCAAGGTTAAAACTCAAATGAATTGACGGGGGCCCGCACAAGCGGTGGAACATGTGGTTTAATTCGATGCAACGCGAAGAACCTTACCTACTCTTGAATCTACAGAAACCGGAA |
| 2-6 | *Halomonas alkaliantarctica* | 97.15% | GGCGTGCGCGGACTTTACTTGCATTTCGAGCGGTTCAGGGGTAGCTTGCTACCCGCTGACGAGCGGCGGACGGGTGAGTAATGCATAGGAATCTGCCCGGTAGTGGGGGATAACCTGGGGAAACCCAGGCTCTTACCGCATACGTCCTACGGGAGAAAGGGGGCTTCGGCTCCCGCTATTGGATGAGCCTATGTCGGATTAGCTAGTTGGTGAGGTAATGGCTCACCAAGGCGACGATCCGTAGCTGGTCTGAGAGGATGATCAGCCACATCGGGACTGAGACACGGCCCGAACTCCTACGGGAGGCAGCAGTGGGGAATATTGGACAATGGGCGGAAGCCTGATCCAGCCATGCCGCGTGTGTGAAGAAGGCCTTCGGGTTGTAAAGCACTTTCAGCGAGGAAGAACGCCTAGTGGTTAATACCCATTAGGAAAGACATCACTCGCAGAAGAAGCACCGGCTAACTCCGTGCCAGCAGCCGCGGTAATACGGAGGGTGCGAGCGTTAATCGGAATTACTGGGCGTAAAGCGCGCGTAGGTGGCTTGATAAGCCGGTTGTGAAAGCCCCGGGCTCAACCTGGGAACGGCATCCGGAACTGTCAAGCTAGAGTGCAGGAGAGGAAGGTAGAATTCCCGGTGTAGCGGTGAAATGCGTAGAGATCGGGAGGAATACCAGTGGCGAAGGCGGCCTTCTGGACTGACACTGACACTGAGGTGCGAAAGCGTGGGTAGCAAACAGGATTAGATACCCTGGTAGTCCACGCCGTAAACGATGTCGACCAGCCGTTGGGTGCCTAGCGCACTTTGTGGCGAAGTTAACGCGATAAGTCGACCGCCTGGGGAGTACGGCCGCAAGGTTAAAACTCAAATGAATTGACGGGGGCCCGCACAAGCGGTGGAGCATGTGGTTTAATTCGATGCAACGCGAAGAACCTTACCTACTCTTGACATCTACAGAAGCCGGAAGAGATTCTGGTGTGCCTTCGGAACTGTAAGACAGTGCTGCATGGCTGTCGTCAGCTCGTGTTGTGAAATGGTGGTTTAGTCCCGAACGAGCCGCA |
| 2-7 | *Halomonas alkaliantarctica* | 96.94% | CCTCACGGCGGCCTACACATGCAAGTCGAGCGGTAACAGGGGTAGCTTGCTACCCGCTGACGAGCGGCGGACGGGTGAGTAATGCATAGGAATCTGCCCGGTAGTGGGGGATAACCTGGGGAAACCCAGGCTAATACCGCATACGTCCTACGGGAGAAAGGGGGCTTCGGCTCCCGCTATTGGATGAGCCTATGTCGGATTAGCTAGTTGGTGAGGTAATGGCTCACCAAGGCGACGATCCGTAGCTGGTCTGAGAGGATGATCAGCCACATCGGGACTGAGACACGGCCCGAACTCCTACGGGAGGCAGCAGTGGGGAATATTGGACAATGGGCGGAAGCCTGATCCAGCCATGCCGCGTGTGTGAAGAAGGCCTTCGGGTTGTAAAGCACTTTCAGCGAGGAAGAACGCCTAGTGGTTAATACCCATTAGGAAAGACATCACTCGCAGAAGAAGCACCGGCTAACTCCGTGCCAGCAGCCGCGGTAATACGGAGGGTGCGAGCGTTAATCGGAATTACTGGGCGTAAAGCGCGCGTAGGTGGCTTGATAAGCCGGTTGTGAAAGCCCCGGGCTCAACCTGGGAACGGCATCCGGAACTGTCAAGCTAGAGTGCAGGAGAGGAAGGTAGAATTCCCGGTGTAGCGGTGAAATGCGTAGAGATCGGGAGGAATACCAGTGGCGAAGGCGGCCTTCTGGACTGACACTGACACTGAGGTGCGAAAGCGTGGGTAGCAAACAGGATTAGATACCCTGGTAGTCCACGCCGTAAACGATGTCGACCAGCCGTTGGGTGCCTAGCGCACTTTTGTGGCGAAGTTAACGCGATAAGTCGACCGCCTTGGGGAGTACGGCCGCAAGGTAAAACTCAAATGAATTGACGGGGGCCCGCACAAGCGGGGGAGCATGTGGTTTAATTCGATGCAACGCGAAGAACCTTACCTACTCTTGAATCTACGGAAGCCGGAAGAGATCTGGTGGGCTTCGGAACTGAAAACAGGTGCTGCTGG |
| 2-8 | *Halomonas qaidamensis* | 99.36% | CCTGACGAGGCTACACATGCAAGTCGAGCGGTAACAGGGGTAGCTTGCTACCCGCTGACGAGCGGCGGACGGGTGAGTAATGCATAGGAATCTGCCCGGTAGTGGGGGATAACCTGGGGAAACCCAGGCTAATACCGCATACGTCCTACGGGAGAAAGGGGGCTTCGGCTCCCGCTATTGGATGAGCCTATGTCGGATTAGCTAGTTGGTGAGGTAATGGCTCACCAAGGCGACGATCCGTAGCTGGTCTGAGAGGATGATCAGCCACATCGGGACTGAGACACGGCCCGAACTCCTACGGGAGGCAGCAGTGGGGAATATTGGACAATGGGCGGAAGCCTGATCCAGCCATGCCGCGTGTGTGAAGAAGGCCTTCGGGTTGTAAAGCACTTTCAGCGAGGAAGAACGCCTAGTGGTTAATACCCATTAGGAAAGACATCACTCGCAGAAGAAGCACCGGCTAACTCCGTGCCAGCAGCCGCGGTAATACGGAGGGTGCGAGCGTTAATCGGAATTACTGGGCGTAAAGCGCGCGTAGGTGGCTTGATAAGCCGGTTGTGAAAGCCCCGGGCTCAACCTGGGAACGGCATCCGGAACTGTCAAGCTAGAGTGCAGGAGAGGAAGGTAGAATTCCCGGTGTAGCGGTGAAATGCGTAGAGATCGGGAGGAATACCAGTGGCGAAGGCGGCCTTCTGGACTGACACTGACACTGAGGTGCGAAAGCGTGGGTAGCAAACAGGATTAGATACCCTGGTAGTCCACGCCGTAAACGATGTCGACCAGCCGTTGGGTGCCTAGCGCACTTTGTGGCGAAGTTAACGCGATAAGTCGACCGCCTGGGGAGTACGGCCGCAAGGTTAAAACTCAAATGAATTGACGGGGGCCCGCACAAGCGGTGGAGCATGTGGTTTAATTCGATGCAACGCGAAGAACCTTACCTACTCTTGACTCTACAGAAGCCGAAAAGAATCTGGGGTGCCTTCGGAACTGTAAACAGGGGCT |
| 2-10 | *Halomonas qaidamensis* | 99.21% | CATGCAAGTCGAGCGGTAACAGGGGTAGCTTGCTACCCGCTGACGAGCGGCGGACGGGTGAGTAATGCATAGGAATCTGCCCGGTAGTGGGGGATAACCTGGGGAAACCCAGGCTAATACCGCATACGTCCTACGGGAGAAAGGGGGCTTCGGCTCCCGCTATTGGATGAGCCTATGTCGGATTAGCTAGTTGGTGAGGTAATGGCTCACCAAGGCGACGATCCGTAGCTGGTCTGAGAGGATGATCAGCCACATCGGGACTGAGACACGGCCCGAACTCCTACGGGAGGCAGCAGTGGGGAATATTGGACAATGGGCGGAAGCCTGATCCAGCCATGCCGCGTGTGTGAAGAAGGCCTTCGGGTTGTAAAGCACTTTCAGCGAGGAAGAACGCCTAGTGGTTAATACCCATTAGGAAAGACATCACTCGCAGAAGAAGCACCGGCTAACTCCGTGCCAGCAGCCGCGGTAATACGGAGGGTGCGAGCGTTAATCGGAATTACTGGGCGTAAAGCGCGCGTAGGTGGCTTGATAAGCCGGTTGTGAAAGCCCCGGGCTCAACCTGGGAACGGCATCCGGAACTGTCAAGCTAGAGTGCAGGAGAGGAAGGTAGAATTCCCGGTGTAGCGGTGAAATGCGTAGAGATCGGGAGGAATACCAGTGGCGAAGGCGGCCTTCTGGACTGACACTGACACTGAGGTGCGAAAGCGTGGGTAGCAAACAGGATTAGATACCCTGGTAGTCCACGCCGTAAACGATGTCGACCAGCCGTTGGGTGCCTAGCGCACTTTGTGGCGAAGTTAACGCGATAAGTCGACCGCCTGGGGAGTACGGCCGCAAGGTTAAACTCAAATGAATTGACGGGGGCCCGCACAAGCGGTGGAGCATC |
| 2-11 | *Halomonas hydrothermalis* | 99.33% | AGTCGAGCGGTAACAGGGGTAGCTTGCTACCCGCTGACGAGCGGCGGACGGGTGAGTAATGCATAGGAATCTGCCCGGTAGTGGGGGATAACCTGGGGAAACCCAGGCTAATACCGCATACGTCCTACGGGAGAAAGGGGGCTTCGGCTCCCGCTATTGGATGAGCCTATGTCGGATTAGCTAGTTGGTGAGGTAATGGCTCACCAAGGCGACGATCCGTAGCTGGTCTGAGAGGATGATCAGCCACATCGGGACTGAGACACGGCCCGAACTCCTACGGGAGGCAGCAGTGGGGAATATTGGACAATGGGCGGAAGCCTGATCCAGCCATGCCGCGTGTGTGAAGAAGGCCTTCGGGTTGTAAAGCACTTTCAGCGAGGAAGAACGCCTAGTGGTTAATACCCATTAGGAAAGACATCACTCGCAGAAGAAGCACCGGCTAACTCCGTGCCAGCAGCCGCGGTAATACGGAGGGTGCGAGCGTTAATCGGAATTACTGGGCGTAAAGCGCGCGTAGGTGGCTTGATAAGCCGGTTGTGAAAGCCCCGGGCTCAACCTGGGAACGGCATCCGGAACTGTCAAGCTAGAGTGCAGGAGAGGAAGGTAGAATTCCCGGTGTAGCGGTGAAATGCGTAGAGATCGGGAGGAATACCAGTGGCGAAGGCGGCCTTCTGGACTGACACTGACACTGAGGTGCGAAAGCGTGGGTAGCAAACAGGATTAGATACCCTGGTAGTCCACGCCGTAAACGATGTCGACCAGCCGTTGGGTGCCTAGCGCACTTTGTGGCGAAGTTAACGCGATAAGTCGACCGCCTGGGGAGTACGGCCGCAAGGTTAAAACTCAAATGAATTGACGGGGGCCCGCACAAGCGGTGGAGCATGTGGTT |
